# Maxillary Sinus Floor Elevation and Simultaneous Implant Installation via Osseodensification Drills: A Retrospective Analysis of Bone Gain in 72 Patients Followed for 6 Months

**DOI:** 10.3390/jcm13082225

**Published:** 2024-04-11

**Authors:** Alper Saglanmak, Ihsan Caglar Cinar, Mohammed Zboun, Volkan Arisan, Eitan Mijiritsky

**Affiliations:** 1 Department of Oral Implantology, Faculty of Dentistry, Istanbul University, Fatih, Istanbul 34093, Türkiye; cinarcaglar@istanbul.edu.tr (I.C.C.); varisan@istanbul.edu.tr (V.A.); 2 Department of Oral & Maxillofacial Surgery and Periodontology, Faculty of Dentistry, Arab American University, 13 Zababdeh, Jenin 240, Palestine; mohammed.alzboun@aaup.edu; 3 Department of Head and Neck Surgery and Maxillofacial Surgery, Tel-Aviv Sourasky Medical Center, School of Medicine, Tel Aviv University, Tel Aviv 64239, Israel; mijiritsky@bezeqint.net; 4 Goldschleger School of Dental Medicine, Faculty of Medicine, Tel Aviv University, Tel Aviv 39040, Israel

**Keywords:** osseodensification, residual bone height, endo sinus bone gain

## Abstract

**Background/Objectives**: The aim of this retrospective study was to radiographically evaluate the endo-sinus bone gain (ESBG) following osseodensification procedures using CBCT and compare the results to more conventional sinus lifting techniques. **Methods**: A total of 72 patients underwent crestal sinus floor elevation procedures and were provided with 102 implants with a sand-blasted and acid-etched surface with microthreads (Medentika^®^ Microcone Implants, Hugelsheim, Germany). Patients were divided into two groups; the osseodensification group (OD; *n* = 36) and the osseodensified augmentation group (ODA; *n* = 36). **Results**: The mean residual bone height (RBH) was 5.71 (1.77) and 4.30 (0.94) mm in the OD and ODA groups, respectively. An ESBG of 3.45 (1.18) and 5.74 (1.31) mm was observed in the OD and ODA groups, respectively, and as compared to the baseline RBH, the ESBG was statistically significant in both groups after 6 months (*p* < 0.001). **Conclusions**: Within the limits of this retrospective study, crestal sinus lifting with the osseodensification technique seems to be a fast, effective, and safe method. Longer follow-up studies with full intrasinus bone topography and structure analyses are needed to prove the success rate of endo-sinus bone gain.

## 1. Introduction

Maxillary posterior edentulism can be challenging for clinicians due to low bone density and the resorption of the alveolar bone by sinus pneumatization [1]. The lateral sinus technique is the first method of choice for an extremely resorbed posterior maxillary region. However, researchers have turned to alternative techniques in order to avoid opening wide flaps, the risk of membrane perforations, the possibility of damage to blood vessels, early and late infection risks, and the need for a serious amount of grafts [2]. Tatum and Summers first described the controlled fracture and elevation of the sinus floor by creating microcracks in the trabecular bone (Summers, 1994) [3,4]. Problems with this approach, such as limited vertical gain (3–4 mm) [5], bone irregularities at the base of the sinus, and trauma to the cranial bones [6], led researchers to develop different, non-invasive techniques, such as minimal invasive antral membrane balloon elevation (MIAMBE) [7], piezo surgery [8], and hydraulic pressure [9].

Recently, a new method called *‘osseodensification’* has become popular. It provides a safe and effective means for transalveolar crestal augmentation. In this technique, a controlled perforation of the sinus floor is achieved using particularly designed burs that rotate counterclockwise, followed by an upward elevation of the Schneiderian membrane using hydraulic pressure [10]. The most important advantages of this technique are reduced surgical time and trauma, clear direct visualization of the membrane, no loss of primary stability, and low risk of membrane perforation [11,12]. In addition to this technique, a new protocol called *‘osseodensified augmentation*’ has been developed in recent years, which combines osseodensification and viscoelastic graft material simultaneously, and provides vertical bone gain that is as significant as the lateral approach [13].

The aim of this retrospective study was to radiographically evaluate the endo-sinus bone gain following the ‘*osseodensification*’ and ‘*osseodensified augmentation*’ procedures using CBCT and compare them to more conventional sinus lifting techniques.

## 2. Materials and Methods

### 2.1. Study Design

Patients who applied to the Istanbul University Department of Oral Implantology between December 2018 and April 2021 with the complaint of maxillary posterior edentulism and received sinus floor elevation treatment using the osseodensification method were included in the study. A total of 72 patients underwent crestal sinus floor elevation procedures and were provided with 102 implants. Using the archived CBCT records of the patients, the residual bone height (RBH) was measured anteriorly and posteriorly at the sites and then averaged accordingly. Patients were divided into two groups; the osseodensification group (OD; *n* = 36) and osseodensified augmentation group (ODA; *n* = 36). A total of 48 implants were placed following the OD protocol and 54 implants were placed following the ODA protocol. Afterwards, implants were divided into three subgroups according to the initial residual bone height (RBH) to determine the correlation with endo-sinus bone gain (ESBG). While the OD group only received the osseodensification procedure, graft augmentation was performed with the osseodensification procedure in the ODA group. This study was conducted in accordance with the World Medical Association Declaration of Helsinki and approved by the ethical committee of the Istanbul University, Faculty of Dentistry (2022/609). Written informed consent was obtained from all patients.

### 2.2. Sample Size Calculation

The sample size was calculated according to the ESBG data and was scrutinized from a prior study in the literature [14]. Considering alpha = 5%, power = 90% with 1.1 mm mean difference between groups, it was calculated that each group should have at least 35 patients.

### 2.3. Patient Selection Criteria

Records of the patients who underwent dental implant treatment in the Department of Oral Implantology, Istanbul University between the dates December 2018 and April 2021 were accessed.

The following inclusion and exclusion criteria were applied: patients with a complaint of tooth loss in the posterior of the maxilla who underwent dental implant treatment using the osseodensification technique were included. To receive the OD protocol that is routinely applied in our clinic, a minimum residual bone height of 5 mm and minimum alveolar width of 4 mm are required. In the same way, to receive the ODA protocol, a minimum residual bone height of 3–4 mm and minimum alveolar width of 5 mm are required. Depending on how much bone elevation is desired, the appropriate protocol is selected. Patients younger than 18 years of age, patients with a diagnosis of an acute sinus infection and/or pathology, patients who received previous sinus augmentation using techniques other than the presently investigated osseodensification technique, and patients with systemic disorders (uncontrolled diabetes mellitus, oncological diagnosis, patients smoking >10 cigarettes/day) which may interfere with bone healing and osseointegration were excluded. Also, patients who did not give consent for the analysis of their medical and demographic data and patients with missing a baseline tomographic image were excluded.

The records of 186 patients who underwent dental implant treatment via the OD or ODA techniques at the Department of Oral Implantology, Faculty of Dentistry, Istanbul University consecutively between the dates December 2018 and April 2021 were accessed and 73 records excluded due to the reasons mentioned above. The accessing of the patients’ files continued until the completion of the required sample size, which was previously determined to be 35. The numeric aims were exceeded to compensate for a possible drop-out due to an unforeseeable data incident. Finally, a total of 72 patient records consisting of 36 OD cases (with 48 implants) and 36 ODA cases (with 54 implants) were included into the final analysis.

### 2.4. Surgical Technique

**Figure 1:**
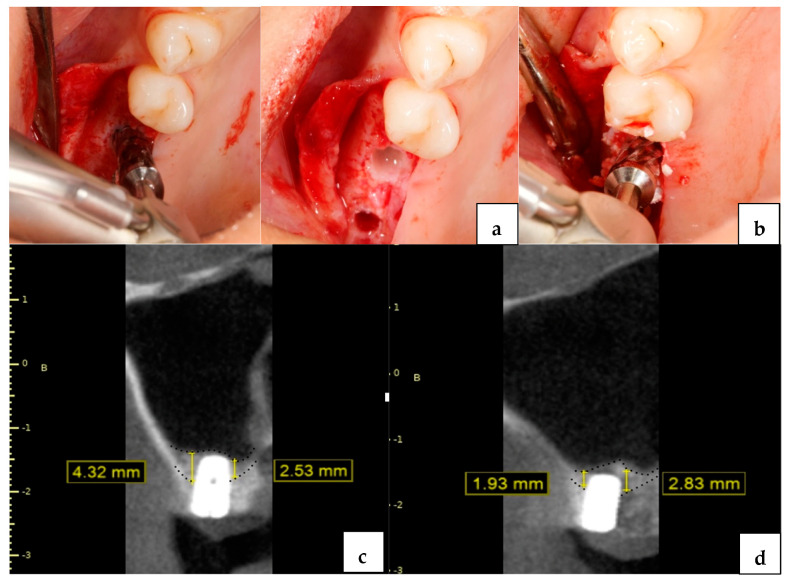
Osseodensification drilling technique in posterior area (**a**); osseodensified augmentation application (**b**); endo−sinus bone gain 6 months post op in ODA Group (**c**); endo−sinus bone gain 6 months post op in OD Group (**d**).

All surgical procedures were performed by two oral surgeons (AS, ICC). The protocol to be followed was decided by the surgeon after CBCT examination. The drilling protocol was performed according to the written protocol of the dedicated manufacturer (Versah, Jackson, MI, USA). Following local anesthetic application, a full thickness flap was raised, and the pilot drill was used to a depth of 1 mm beneath the sinus floor (clockwise rotation 1200 rpm). In the osseodensification group (OD Group), sequential drilling was performed with a 2.0 drill in a counterclockwise rotation (1200 rpm) with copious irrigation until haptic feedback was felt at the sinus floor. With successive 3.0 and 4.0 burs, a bouncing motion with gentle pressure was applied until the sinus floor was perforated, and the sinus membrane was lifted up to 3 mm with slight gradual progressions before the implant installment (Figure 1).

In the osseodensified augmentation group (ODA Group), no pilot burs were used, and the subantral bone was drilled using 2.0 and 3.0 burs, respectively (Counterclockwise rotation 1200 rpm with copious irrigation). The cortical layer was perforated with 4.0 and 5.0 densah burs and the membrane was lifted 3 mm. Subsequently, a viscoelastic colloidal graft material (Osteobiol^®^ putty graft, Pianezza, Italy) was applied into the osteotomy cavity with the help of the final drill (100 RPM counterclockwise rotation without irrigation). The grafting step was repeated until the desired membrane elevation was achieved (Figure 1). In the OD and ODA groups implants, sand-blasted and acid-etched surfaces with microthreads (Medentika^®^ Microcone Implants, Hugelsheim, Germany) were placed using the surgical hand piece with 15 RPM and a maximum insertion torque value of 45 N/cm. All submerged implants were left to heal for 6 months. All implants were rehabilitated with a screw retained prosthesis. The integrity of the Schneiderian membrane was routinely checked with CBCT images taken after the surgical procedure.

### 2.5. Outcome Measures

For the calculation of the endo-sinus bone gain (ESBG), all measurements were performed on a personal computer, using a dedicated software (DTX Studio™ Implant 3.4.3.3, Nobel Biocare AG). On the baseline CBCT image, the cortical contour at the inferior part of the maxillary sinus was marked. The residual bone height (RBH) was measured from the mesial and distal walls of the determined implant recipient site. Then, at the post-op 6 months following the CBCT, the site was reopened, and the cross-section was aligned at the center of the long axis of the placed implant and double-checked with the residual bone height (RBH), which was measured preoperatively. On the mesial and distal CBCT cross-section, the distance between the marginal crest and the apex of the implant was measured. The bone margin visible at the apex of the implant was assumed to be achieved by the OD or ODA procedures. The endo-sinus bone gain (ESBG) distance was calculated by extracting the RBH from the distance between the marginal crest and the apex of the implant. All measurements were in the unit of millimeters.

### 2.6. Statistical Analysis

The statistical analysis was performed with a commercially available software program (Stata 17.0 MP Parallel Edition, Stata Corp., College Station, TX, USA). Descriptive statistics, including mean and standard deviation, were carried out. The Kolmogorov–Smirnov analysis was used to check the normality of the distribution of the data. The similarity of the baseline cohort characteristics including, age, gender, RBH, and the diameter and the length of placed implants were examined using the Chi-square test. The unpaired *t* test was used for evaluating the RBH and ESBG between groups. The paired sample t test was used for in time group evaluations. The relationship between the RBH and ESBG was analyzed using Pearson correlation analysis. The significance level was set as *p* < 0.05.

## 3. Results

A total of 72 patients (33 males and 39 females with a mean age of 53.26 ± 10.76) receiving 102 implants placed concomitantly with the sinus floor elevation procedure using an osseodensification protocol were examined radiographically. The mean overall RBH was 4.96 ± 1.55 mm. A total of 36 patients with 48 implants in the OD group and 36 patients with 54 implants in the ODA group were analyzed. The mean time between the implant installation and the post-op evaluation was 194.6 ± 24.8 days.

### Complications

One patient in the ODA group applied to the clinic following the 10th day of surgery due to severe pain in the area. The examination revealed acute sinusitis, and Levofloxacin (Tavanic 500 mg 1 × 1, for 14 days; Sanofi-Aventis, Paris, France) with a nasal decongestant including xylometazoline HCl (Otrivine care 1 mg/mL; Glaxosmithkline, London, UK) was prescribed. The complaints were resolved, and no further symptoms were recorded. Epistaxis was observed in one patient of the OD group for the first two days after surgery. The epistaxis resolved naturally with no further complaints. No further complications were recorded in any of the patients.

The number of patients, number of sites, age and gender of patients, RBH, and implant diameter and length associated with the study groups are presented in Table 1. The baseline characteristics of the study populations were similar in terms of age, gender, and implant size characteristics (*p* > 0.05).

The mean RBH was 5.71 (1.77) and 4.30 (0.94) mm in the OD and ODA groups, respectively. An ESBG of 3.45 (1.18) and 5.74 (1.31) mm was observed in the OD and ODA groups, respectively, and as compared to the baseline RBH, the amount of ESBG was statistically significant in both groups after 6 months (*p* < 0.001). After 6 months, the differences in ESBG between the groups were statistically significant (*p* < 0.001) (Table 2).

A negative correlation was observed between the RBH and ESBG in both groups (*p* < 0.001) (Table 3 and Table 4).

## 4. Discussion

In this retrospective study, the osseodensification method was used to simultaneously augment the maxillary sinus and place implants in the posterior maxilla of patients with reduced residual bone height. Sinus floor elevation using the osseodensification technique is a fast, effective, and reliable technique that has been used frequently in recent years [15]. Specially designed burs and their counterclockwise rotations allow for high tactile sensation and prevent sinus membrane perforations despite working at relatively high speeds. Also, the pumping action of the bur, accompanied with hydraulic pressure and viscoelastic colloidal graft material, allow for the easy and gentle lifting of the Schneiderian membrane [16]. In addition, increased bone density around the osteotomy—owing to the fact that the lateral and apical compaction of the autograft fragments come out (displaced) during osteotomy preparation with these burs—enhances implant primary stability and increases the initial BIC [17,18].

A desirable level of membrane elevation was achieved in both OD and ODA groups, even in decreased RBH cases. A highly statistically significant difference was found in terms of ESBG when compared to each other and in time group evaluations. At the 6-month time point, the OD and ODA groups showed an average ESBG of 3.45 and 5.74 mm, respectively. These findings, as summarized in Table 2, are correspondent with similar results obtained in another study by Neiva et al. (i.e., 2.8 mm ESBG with OD and 5.9 mm ESBG with ODA protocol) [13]. Additionally, there were negative correlations between RBH and ESBG in the study groups. Taking into consideration the simultaneous implant placement, such findings are comprehensible as a forgone conclusion; a lower RBH requires a greater bone graft volume to achieve the same level of membrane elevation and augmentation. Promisingly, it seems possible to simultaneously place standard length implants even in reduced RBH cases with or without augmentation, which is purely due to the favorably high level of membrane elevation and ESBG to be achieved. A study conducted by Puterman et al. demonstrated how clearly the Schneiderian membrane is visualized through the osteotomy, particularly in reduced RBH cases [15]. The membrane is seen apically just beyond the point where the sub-sinus cortical perforation takes place.

In relation to endo-sinus bone regeneration, two biological concepts were raised. One concept interprets the new bone formation as a result of osteogenic progenitors derived from the surrounding anatomical structures such as the bone marrow stroma, periosteum, and microvascular walls [19]. The sinus membrane itself has a potential role in bone formation as it contains mesenchymal progenitor cells and cells with an active role in the osteogenic lineage [20]. The concept gives more credit to the sinus membrane, which is capable of functioning as a barrier and maintaining the blood clot in the space created after the membrane elevation [21]. The implants’ role is to hold the membrane elevated up on the apex of the implant. In turn, the fibrin clot promotes the osteogenesis process. Such endo-sinus bone gain was noticed in clinical studies, even though no bone graft material was used in cases of synchronous implant installments with membrane elevation [14,22].

Palma et al. evaluated the relationship between new bone formation and the elevated sinus membrane histologically—without any grafting materials—and the results showed that the newly formed trabecular bone at the implant apex was lining up with the Schneiderian membrane. This phenomenon was also detected in the OD group [22]. In their study, Chen et al. were oriented to the lateral window access to achieve a highly tended Schneiderian membrane to create space for bone regeneration and maintenance by simultaneous implant placement [23]. Therefore, the higher the protrusion length of the installed implants into the sinus space, the higher the bone regeneration in the region; the reason is that the implants serve as tenting poles, which in turn maintain the level of the membrane as high as they are lifted [14,20,24]. In the same manner, Lai et al. did find a significant correlation between implant protrusion length and ESBG at the end of the 9-month follow-up period in patients with osteotome sinus floor elevation (OSFE) [25].

Many studies using the crestal approach have mentioned residual bone height as a predictor for implant survival [26,27]; consequently, using such an approach exclusively in cases with ≥5 mm residual subcantral bone height is recommended. Nedir et al. adopted the OSFE (osteotome sinus floor elevation) protocol without grafting in their study, in which a mean ESBG of 2.5 ± 1.2 mm after 1 year was obtained. Yet, this remains clinically low when compared to the OD protocol in the present study. Moreover, 16% of the cases encountered perforation of the Schneiderian membrane as well [28]. Other studies in which the OSFE protocol was implemented have confronted the high tendency of perforation in cases with minimal RBH [29]. The disruption of the integrity of the Schneiderian membrane can lead to infection and implant failure [30]. Many studies have indicated that an increasing rate of membrane perforation coincided with a reduced RBH after the OSFE protocol was applied [31], although some studies still consider such perforations clinically insignificant [28,32]. On the contrary, membrane perforations were not encountered in this study or in other similar retrospective clinical studies [10] and/or case reports [2]. The special design of the osseodensification burs, together with the hydraulic pressure that effectively releases the Schneiderian membrane off the sinus floor, seem to critically decrease the tension in the membrane, thus preventing perforation and allowing for significantly higher membrane elevation and ESBG values, even in decreased RBH cases. Some researchers recommend using the final drill to perform the cortical perforation, as the wider the drill tip is, the lesser the risk of any perforation of the membrane or bleeding that may impair clear visualization, all of which is believed to be attributed to the broader distribution of the force applied by the drill [15]. In the present study, differing intermediate burs were used to travel through (traverse) the sinus floor, and still, no perforations were encountered. The “haptic feedback” tactile sensation coupled with the tapering design of the osseodensification drills allows for maximum control at this critical stage. Thereafter, propelling the viscoelastic graft material through the osteotomy was carried out using the densifying feature of the drills. The graft material passed through the osteotomy into the maxillary sinus where the Schneiderian membrane distended further up while the graft was spreading evenly around the working end of the burs.

In their study, Berengo et al. performed an intraoperative endoscopic evaluation of OSFE, where they found that crestal augmentation was predictable. Additionally, low rates of perforations and complications were observed in cases in which the raised sinus membrane showed lateral distension [32]. The lateral distension of the membrane is possible where the undermined sinus membrane detaches further into the periphery of the created subantral space. In cases where the base was broadened, perforation rates were low and crestal sinus lifting was desirable. This finding was in accordance with other studies [28,33]. According to Pommer et al., the perforation of the sinus membrane occurs at a mean tension force value of 7.3 N/mm [34]. It was mentioned that membrane perforations were caused by two main factors, which were bone resistance at the base of the sinus and the applied forces with instruments during the augmentation of the sinus [33]. In the present study, hydrostatic pressure during drilling and the incremental application of the viscoelastic colloidal graft material during grafting were imperative to prevent undue tension and pressure on the Schneiderian membrane and led it to show lateral distension. This raises the questions of how evenly the graft material is distributed and how to interpret the modality of the detached membrane repositioning around the placed implants. The viscoelastic properties of the graft material allow for an even peripheral detachment of the membrane during the graft application, creating a wide based dome-like shape. Moreover, the demarcation point of the grafted area can easily be traced on radiographic images obtained pre- and post-operatively, owing to the radiopacity of the graft material. Not only does this radiopacity allow for the clear visualization of the progress of the material intraoperatively, but it also makes it possible to analyze changes occurring through time during follow-up visits. A slight collapse of the formed dome-like grafted mass during the early remodeling phase is of decisive importance for the long-term outcome. Therefore, this prospect, and how much of the ESBG is preserved in the long-term, must be measured and evaluated in more extended and well-designed studies. Also, it would be of interest to yield the measurements of the protruded portion of the implants in relation to the RBH, supplemented with evaluations of the morphology of the newly formed bone around the installed implants [35].

Another concern related to the conventional closed approach is that the osteotomes used are believed to cause undue fractures at the trabecular level that require longer healing times, thus delaying the onset of secondary stability [36]. Osseodensification drills maintain the autologous osseous densification feature of traditional osteotomes by expanding the osteotomy with a sliding and rolling contact without causing associated damage at the trabecular level [37,38]. The spring-back effect of the condensed bone more than likely ensures primary stability. It would be interesting to compare implant stability metrics, early bone healing histology, and the change in ESBG values over time around implants placed using the OSFE and osseodensification protocols. Our protocol did not include regular stability measurements such as insertion torque (IT) or resonance frequency analysis (RFA) at predetermined time points.

In cases where more than 3 mm of ESBG is required for implant placement, the preferred conventional approach is the lateral window technique [39], and in cases where the residual bone height is ≤ 4 mm, a two-stage approach is recommended. In a series of studies that compared one-stage/two-stage implant placement procedures in low RBH cases (<4 mm), there was no statistical difference in 10-year cumulative survival rates (96.8% and 92.5%, respectively) [40]. Subsequently, the choice here is centered around the question of primary stability, since it is well known that bone density is a strong predictor of implant primary stability [41]. Osseodensification drills compact and lateralize autologous bone and potentially increase the initial mechanical stability around simultaneously placed implants [42], thereby achieving a stable implant placement even in reduced RBH cases, with or without a graft placement. Again, in the present study, implant placement was feasible in all the cases with RBH ranges between 2 and 4 mm following either OD or ODA protocols. Considering the extensive flaps, technical sensitivity, and complication rates of traditional lateral window sinus lifting procedures, the less invasive trans alveolar lift protocol via osseodensification is destined to be highly regarded, even where implant placement is not likely [2]. 

## 5. Conclusions

Within the limits of this retrospective study, crestal sinus lifting with the osseodensification technique seems to be a fast, effective, and safe method. Unlike other techniques used to create a subantral space, osseodensification can be considered a predictable modality even in cases with low RBH. To validate the feasibility of these techniques, longer follow-up studies with full intrasinus bone topography and structure analyses are needed to prove the success rate of endo-sinus bone gain via the osseodensification technique performed with or without grafts, as compared to conventional sinus lifting techniques.

## Figures and Tables

**Table 1:** Descriptive summary of study sample.

	*p* Value	OD	ODA
Patient		36	36
Sites		48	54
Demographic Variable			
Age < 50	0.137	22	17
Age > 50	0.092	26	37
Female		27	39
Male		21	15
Site Related Variables			
RBH (mm)	<0.00001 *	5.71 ± 1.77	4.30 ± 0.94
Diameter	0.179		
4.0		27	21
5.0		21	33
Length (mm)			
8	0.756	22	21
9		15	18
11		11	15

Note: Data are presented as number of sites. Chi square test for categorical variables. *P* value: “*” indicates *p* < 0.001.

**Table 2:** Endo-sinus bone gain according to study groups at 6 months.

	OD	ODA	*p* Value
RBH Mean	5.71 ± 1.77	4.30 ± 0.94	<0.00001 *
Subject Number	48	54	
ESBG (mm)	3.45 ± 1.18	5.74 ± 1.31	<0.00001 *
*p* Value	<0.00001 *	<0.00001 *	

Level of significance set at *p* < 0.05; pnpaired *t* test for study groups; paired sample *t* test for in time group evaluations. *P* value: “*” indicates *p* < 0.001.

**Table 3:** Endo-sinus bone gain according to RBH at 6 months after osseodensification protocol (OD).

	RBH (2–4 mm)	RBH (4–6 mm)	RBH (6–9 mm)
Mean	3.70 ± 0.20	5.34 ± 0.27	8.45 ± 0.57
Subject Number	12	24	12
ESBG (mm)	4.55 ± 0.15	3.26 ± 0.56	2.75 ± 1.82
Correlation Coefficient	−0.6095		
*p* Value	<0.00001 *		

Level of significance set at *p* < 0.05; Pearson correlation analysis. *P* value: “*” indicates *p* < 0.001.

**Table 4:** Endo-sinus bone gain according to RBH at 6 months after osseodensified augmentation protocol (ODA).

	RBH (2–4 mm)	RBH (4–6 mm)	RBH (6–9 mm)
Mean	3.37 ± 0.32	4.75 ± 0.55	6.30 ± 0.10
Subject Number	21	30	3
ESBG (mm)	6.37 ± 1.61	5.46 ± 0.85	4.15 ± 0.19
Correlation Coefficient	−0.5043		
*p* Value	<0.001 *		

Level of significance set at *p* < 0.05; Pearson correlation analysis. *P* value: “*” indicates *p* < 0.001.

## Data Availability

The original contributions presented in the study are included in the article, further inquiries can be directed to the corresponding author/s.
